# Effects of methacholine infusion on desflurane pharmacokinetics in piglets^☆^

**DOI:** 10.1016/j.dib.2015.11.002

**Published:** 2015-11-10

**Authors:** Alf Kozian, Moritz Kretzschmar, James E. Baumgardner, Jens Schreiber, Göran Hedenstierna, Anders Larsson, Thomas Hachenberg, Thomas Schilling

**Affiliations:** a Department of Anesthesiology and Intensive Care Medicine, Otto-von-Guericke-University Magdeburg, Germany; b Department of Surgical Sciences, Hedenstierna Laboratory, Uppsala University, Uppsala, Sweden; c Oscillogy ® LLC, Folsom, PA, USA; d Department of Pneumology, Otto-von-Guericke-University Magdeburg, Germany; e Department of Medical Sciences, Clinical Physiology, Uppsala University, Sweden; f Department of Surgical Sciences, Anesthesia and Intensive Care, Uppsala University, Uppsala, Sweden

**Keywords:** Animal model, Alveolar ventilation, Desflurane, Arterial blood gas concentration, Bronchoconstriction, Ventilation-/perfusion-mismatch, Methacholine

## Abstract

The data of a corresponding animal experiment demonstrates that nebulized methacholine (MCh) induced severe bronchoconstriction and significant inhomogeneous ventilation and pulmonary perfusion (V̇_A_/Q̇) distribution in pigs, which is similar to findings in human asthma. The inhalation of MCh induced bronchoconstriction and delayed both uptake and elimination of desflurane (Kretzschmar et al., 2015) [1].

The objective of the present data is to determine V̇_A_/Q̇ matching by Multiple Inert Gas Elimination Technique (MIGET) in piglets before and during methacholine- (MCh-) induced bronchoconstriction, induced by MCh infusion, and to assess the blood concentration profiles for desflurane (DES) by Micropore Membrane Inlet Mass Spectrometry (MMIMS).

Healthy piglets (*n*=4) under general anesthesia were instrumented with arterial, central venous, and pulmonary artery lines. The airway was secured via median tracheostomy with an endotracheal tube, and animals were mechanically ventilated with intermittent positive pressure ventilation (IPPV) with a FiO_2_ of 0.4, tidal volume (*V*_T_)=10 ml/kg and PEEP of 5cmH_2_O using an open system. The determination of V._A_/Q. was done by MIGET: before desflurane application and at plateau in both healthy state and during MCh infusion. Arterial blood was sampled at 0, 1, 2, 5, 10, 20, and 30 min during wash-in and washout, respectively.

Bronchoconstriction was established by MCH infusion aiming at doubling the peak airway pressure, after which wash-in and washout of the anesthetic gas was repeated. Anesthesia gas concentrations were measured by MMIMS. Data were analyzed by ANOVA, paired *t*-test, and by nonparametric Friedman׳s test and Wilcoxon׳s matched pairs test.

We measured airway pressures, pulmonary resistance, and mean paO_2_ as well as hemodynamic variables in all pigs before desflurane application and at plateau in both healthy state and during methacholine administration by infusion. By MIGET, fractional alveolar ventilation and pulmonary perfusion in relation to the V.A/Q. compartments, data of logSDQ̇ and logSDV̇ (the second moments describing global dispersion, i.e. heterogeneity of distribution) were estimated prior to and after MCh infusion. The uptake and elimination of desflurane was determined by MMIMS.

**Specifications Table**

**Table:** 

Subject area	Biochemistry
More specific subject area	Respiratory Physiology, Pharmacokinetics
Type of data	Tables, text file, graph, figure
How data was acquired	Mass Spectrometry, Multiple Inert Gas Elimination Technique
Data format	Calculated and statistically analyzed data in tables and figures
Experimental factors	Respiratory physiology determined before desflurane application and at plateau in both healthy state and during methacholine infusion
Experimental features	Multiple Inert Gas Elimination Technique allows the determination of ventilation and pulmonary perfusion distribution. The uptake and elimination of desflurane in arterial blood was determined by Micropore Membrane Inlet Mass Spectrometry
Data source location	University of Uppsala, Uppsala, Sweden
Data accessibility	Data is provided within this article

**Value of the data**

• The potential changes of uptake and elimination of a volatile anesthetic on the basis of uneven distribution of ventilation and/or pulmonary perfusion have not been demonstrated yet.
• This experimental data provides insights on how the pharmacokinetics of volatile anesthetics is affected by bronchoconstriction and calculated ventilation/perfusion (V̇_A_/Q̇) ratios depending on intravenous administration of methacholine, a potent bronchoconstrictor, in a piglet model.
• The present data are useful in implementing animal pharmacokinetic models for studying uptake and elimination of inhalational anesthetics.
• Data from the present porcine model of bronchoconstriction might be considered when administering inhalational anesthesia to asthmatic patients in clinical studies.

## 1 Data

The data of an animal experiment on the effects of methacholine (MCh) infusion on desflurane (DES) uptake and elimination are shared with this data article. It comprises baseline hemodynamics, gas exchange and ventilation data in healthy piglets, and cardiopulmonary data during MCh infusion. The mean values of hemodynamics, ventilation, and gas exchange prior to and during MCh infusion are displayed in Table 1, Table 2, respectively.

**Fig. 1:**
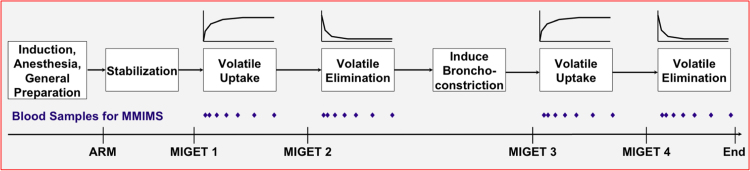
Experimental timeline. ARM Alveolar Recruitment Maneuver, MMIMS Micropore Membrane Inlet Mass Spectrometry, MIGET Multiple Inert Gas Elimination Technique.

The experimental protocol is presented in Fig. 1.

**Fig. 2:**
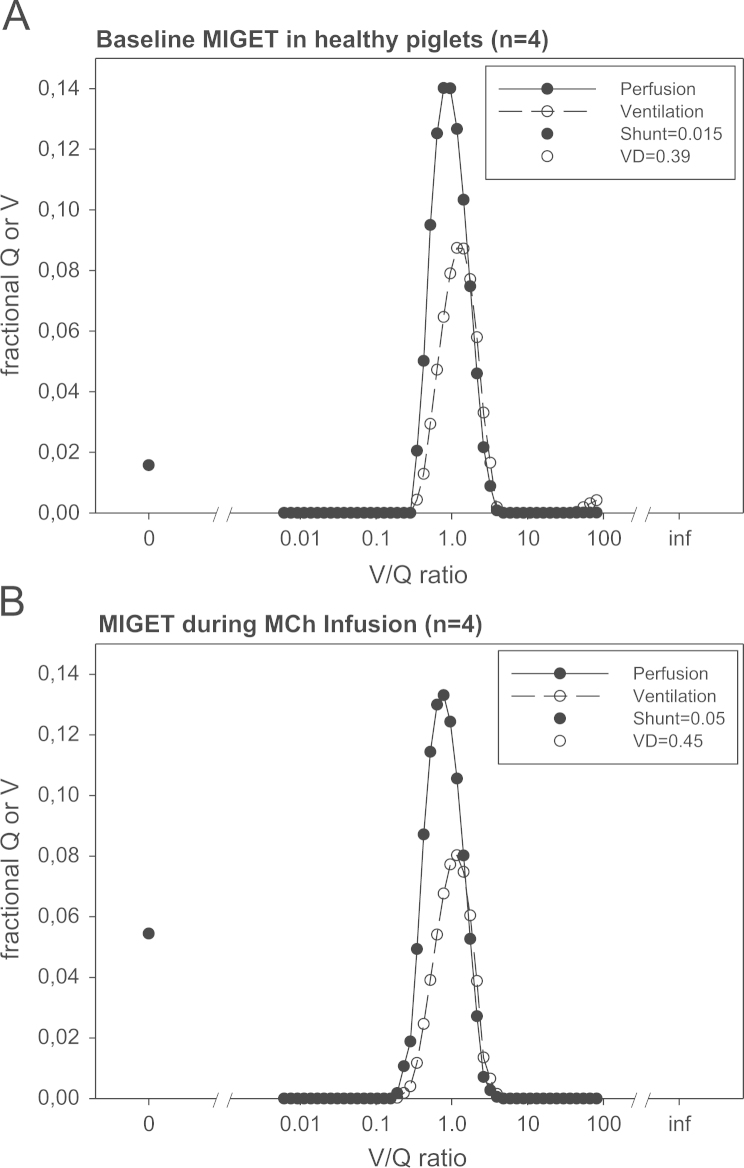
MIGET fractional ventilation (V) and pulmonary perfusion (Q) by different V̇/Q̇ ratios at baseline (A) and during MCh infusion (B). The data represents the averaged moments of ventilation and perfusion distribution of all piglets. Note that there is no difference induced by infusion of MCh in comparison to healthy animals. MCh Methacholine, MIGET Multiple Inert Gas Elimination Technique, Q Pulmonary Perfusion, V Alveolar Ventilation, VD Dead Space.

Multiple Inert Gas Elimination Technique (MIGET) was used to determine alveolar ventilation and pulmonary perfusion distribution (V.A/Q.) before and during MCh infusion. The median values of MIGET variables and IQR are given in Table 3. The fractional alveolar ventilation and pulmonary perfusion in relation to the V.A/Q. compartments is displayed in Fig. 2.

**Fig. 3:**
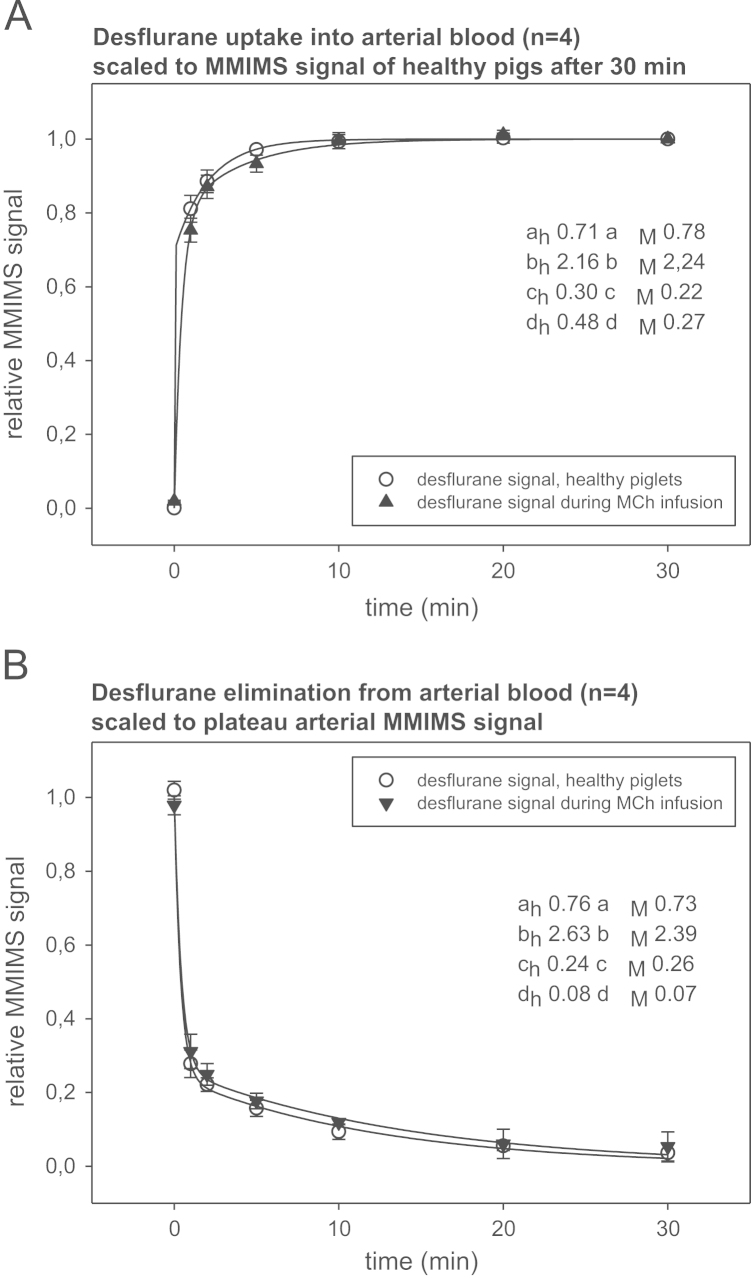
Time course of desflurane uptake (A) into and elimination (B) from arterial blood in healthy (h) and MCh injured piglets (M). Data were calculated as means (SD) after scaling the MMIMS signals in the individual piglet to the arterial plateau after 30 min. The infusion of MCh induced bronchoconstriction and impaired arterial oxygenation but did not affect desflurane pharmacokinetics. Enclosed are the mean coefficients (a, b, c, d) of the exponential regression functions *y*_*u*_=*f*(*x*)=*a*(*1e*^*-bx*^)+*c*(*1*−*e*^−^^*dx*^) and *y*_*e*_=*f*(*x*)=*a*(*e*^*−bx*^)*+c*(*e*^*−dx*^), displayed for uptake (u) and elimination (e) of desflurane. MMIMS Micropore Membrane Inlet Mass Spectrometry, MCh Methacholine, h healthy, M MCh injured.

Desflurane concentrations in arterial blood samples were analyzed by Micropore Membrane Inlet Mass Spectrometry (MMIMS). Data on arterial blood desflurane during uptake and elimination are displayed in Fig. 3. The infusion of MCh induced bronchoconstriction and impaired arterial oxygenation did not affect desflurane pharmacokinetics.

## 2 Experimental design, materials and methods

### 2.1 General

The present experimental design, materials and methods are in accordance with the corresponding experiment on the effects of MCh inhalation on desflurane pharmacokinetics [1]. It was scheduled as a prospective, controlled, animal trial in a single cohort of juvenile piglets. The Animal Ethics Committee of Uppsala University (Sweden) approved the experimental protocol. The care and handling of animals were in accordance with National Institutes of Health guidelines for ethical animal treatment [2].

### 2.2 Animals

Healthy, 2.5-month-old piglets (26.7±0.5 kg) of mixed Yorkshire/Norwegian country breeds obtained from a local breeder were included (*n*=4). The animals fasted overnight with free access to water. All animals underwent the same routine instrumentation and monitoring algorithm including induction and maintenance of intravenous anesthesia.

### 2.3 Anesthesia, instrumentation and monitoring

The animals were anesthetized by an i.m. injection of xylazine (2.2 mg kg^−1^, Rompun®; Bayer, Leverkusen, Germany), and tiletamine/zolazepam (6 mg kg^−1^, Zoletil®; Virbac, Carros, France). The animals were placed in supine position and general anesthesia was continued by infusions of fentanyl (Leptanal®; Janssen-Cilag AB, Sweden; 0.04 mg kg^−1^ h^−1^), midazolam (0.12 mg kg^−1^ h^−1^, Midazolam Actavis, Actavis Group, Hafnersfjordur, Iceland), and propofol (Diprivan®; Astra, Södertälje, Sweden; 6 mg kg^−1^ h^−1^) via 18G catheters (Becton Dickinson, Heidelberg, Germany), placed in ear veins. The absence of consciousness was verified by testing the corneal reflex and the hind limb reflex response, and muscle relaxation was induced with a bolus of rocuronium bromide (2 mg kg^−1^, Esmeron®, N.V. Organon, Oss, Netherlands) followed by a continuous infusion of the same drug at 2.5 mg kg^−1^ h^−1^. The trachea was intubated with an ID 7.0 mm cuffed endotracheal tube (Mallinckrodt, Athlone, Ireland). The animals were mechanically ventilated with intermittent positive pressure ventilation (IPPV) with an inspiration/expiration ratio of 1:2, inspired oxygen fraction (F_I_O_2_) of 0.4 and PEEP of 5cmH_2_O provided by a KION® anesthesia ventilator (Maquet Critical Care, Solna, Sweden). The tidal volume (*V*_T_) was set to 10 ml kg^−1^, and respiratory frequency was adjusted to achieve a normal arterial pCO_2_ of 40 mmHg. Exhaled end-tidal CO_2_ (etCO_2_), gas flow and airway pressures were measured at the proximal end of the tracheal tube with a NICO capnograph (Respironics, Wallingford, CT). A F_I_O_2_ of 0.4 was maintained throughout the experiment.

A 14G catheter was placed in one of the front limbs for MIGET infusion and a flow-directed pulmonary artery catheter (PAC, 7.0 French, Swan-Ganz thermodilution catheter, Baxter, Irvine, CA, USA) and a single lumen central venous catheter (4.0 French, Becton-Dickinson Critical Care Systems, Singapore) were inserted via the right internal jugular vein. The PAC was used for cardiac output measurements and mixed venous blood sampling. Temperature was also measured by PAC and maintained at 37.5±0.5 °C by use of heating mats. The PAC was repositioned frequently to ensure that the tip was always located in regions with high pulmonary blood flow. Hemodynamic parameters (cardiac output (CO), heart rate (HR), mean arterial pressure (MAP), mean pulmonary arterial pressure (MPAP), central venous pressure (CVP) and peripheral capillary oxygen saturation (SpO_2_) were monitored continuously with a SC 9000 XL Monitor (Siemens, Erlangen, Germany) and recorded at each experimental setting.

An arterial catheter was inserted into the right carotid artery for continuous arterial pressure measurements and a hind limb arterial catheter for blood sampling (20G; Becton-Dickinson Critical Care Systems).

Blood gas analysis was performed immediately after bubble free blood sampling with standard blood gas electrodes (ABL 500; Radiometer, Copenhagen, Denmark). The blood gas analyzer was calibrated (one-point every hour and two-points every four hours) by internal routines, and always before the day׳s measurements. Finally, a suprapubic urinary catheter (Sympakath; Ruesch AG, St Gallen, Switzerland) was placed for monitoring of urine output. After completion of general preparation, an alveolar recruitment maneuver (inspiratory hold, 40cmH_2_O, for 10 s) was performed, and the piglets were allowed 30 min for stabilization.

The animals received 10±2 ml kg^−1^ h^−1^ of saline (Fresenius Kabi AB; Halden, Norway). At the end of the experiment, the animals were euthanized with an *i.v.* injection of potassium chloride (150 meq) while under general anesthesia.

### 2.4 Multiple inert gas elimination technique (MIGET)

For MIGET, six inert gases were dissolved in saline and infused into a peripheral vein at a constant rate. Samples of mixed venous blood (input of gases to the lung), systemic arterial blood (gases retained in the lung in blood phase) and mixed expired gas (gases excreted from the lung in gas phase) were analyzed for their inert gas partial pressures. The differences in the way the lung retains or excretes gases of different solubility were analyzed to determine a compatible distribution of the ventilation/perfusion ratios (V._A_/Q.) [3].

The inert gas infusion was saline with added sulfurhexafluoride (SF6), ethane and cyclopropane (by bubbling) and enflurane, diethyl ether and acetone (by liquid injection). This mixture was infused into a peripheral vein at a rate of 3 ml/min. Determination of V._A_/Q. was carried out four times: before desflurane application and at the plateau state after wash-in in both healthy piglets and during methacholine administration (i.e. during bronchoconstriction). Blood samples for MIGET were collected simultaneously from the pulmonary artery and systemic artery catheters in 10 ml gas-tight, ungreased, matched barrel glass syringes (Popper & Sons, now Cadence Science, Staunton, VA, USA) prefilled with heparin. Mixed expired gas samples were collected in dry 20 ml, matched barrel glass syringes from a heated (>40 °C) mixing chamber (volume 2.5 l) connected to the ventilator outlet.

Analysis of the gas samples by gas chromatography (GC, Model 5890, Series II; Hewlett-Packard, Waltham, MA) and calculations were carried out as previously reported [4], [5].

### 2.5 Anesthesia gas measurement by mass spectrometry

Arterial blood samples were collected in glass syringes coated with EDTA (FORTUNA® OPTIMA®, 5 ml, Luer-lock, Poulten&Graf GmbH, Wertheim, Germany) for analysis by Micropore Membrane Inlet Mass Spectrometry (MIGET by MMIMS System, Oscillogy®, Folsom, PA, USA).

For analysis, the blood samples flowed over the MMIMS probes and the volatile gas partial pressures were analyzed directly. The user entered essential physiologic data (cardiac output, minute ventilation, core body temperature) into the machine, and the system adjusted the probe and sample temperatures to match core temperature. Systemic artery blood samples and mixed expired gas samples were injected into the machine. The system flows each sample over a microporous membrane inlet probe, measures the current signal from the mass spectrometer at m/e=101 for desflurane, identifies the plateau signal and averages that signal [6], [7], [8], [9].

### 2.6 Experimental protocol

(1) Baseline: Following a lung recruitment maneuver (inspiratory hold function of the respirator, 40cmH_2_O, for 10 s) and stabilization period, baseline hemodynamic, ventilation, and gas exchange data were obtained.

(1) Healthy state: Desflurane (Suprane®, Baxter) was administered via the KION ventilator with the vaporizer set at 1 MAC (≈6 vol%) in an open system with fresh gas flow set to exceed double minute ventilation. End-tidal gas samples as well as arterial blood were obtained simultaneously at 0, 1, 2, 5, 10, 20 and 30 min (volatile uptake, wash-in). In a second step, administration of desflurane was stopped and the sampling was repeated (volatile elimination, wash-out).

(1) Induction of bronchoconstriction: This was induced by infusion of methacholine (MCh) (Acetyl-ß-methacholine chloride, Sigma-Aldrich Inc. St. Louis, Mo, USA). Infusion was performed continuously with 100 µg/ml MCh in normal saline at 50–100 ml/h and adjusted to maintain doubling of peak airway pressure in each pig throughout the second part of the experiment. Thereafter, desflurane uptake and elimination measurements were repeated during MCh infusion, using the same timing schedule.

The workflow of the experimental protocol is presented in Fig. 1.

### 2.7 Data analysis

Data analysis was performed with the Statistical Package for the Social Sciences (SPSS, v. 22, IBM Corporation, Armonk, New York, USA) and SigmaPlot® v. 11 (Systat Software Inc., San Jose, CA, USA). The estimation of sample size was based on previous experimental studies, which used an analogous experimental setup [1], [10], [11], [12]. Power calculation using a two-sided design at a significance level of 5% (*α*=0.05) and a power of 80% (*β*=0.20) revealed that at least 4 animals per group were needed to detect a difference of more than 20% in the time to P90 arterial desflurane concentrations during MCh inhalation [1]. This time point was defined as the primary variable.

The data were tested for normal distribution with the Shapiro–Wilks *W* test and are presented as means and standard deviations in the case of normal distribution (cardiopulmonary and ventilation variables). Nonlinear regression analysis of MMIMS data was performed with Sigmaplot®. The curves are displayed as mean of data points with standard deviations.

The analysis of normally distributed data was performed by a repeated measures one-way analysis of variance (ANOVA) with post-hoc Bonferroni correction. The V._A_/Q. distributions were compared after calculations of moments of the distributions (mean V._A_/Q., Vmean, Qmean, logSDV, logSDQ and skewness) by nonparametric Friedman׳s test and Wilcoxon׳s matched pairs test [13].

No data were lost during the experiment or were missed in the statistical analysis. The differences were considered to be statistically significant for all procedures if *p*<0.05.

## Figures and Tables

**Table 1:** Cardiopulmonary variables at baseline, prior to and after desflurane administration (*n*=4). CO, MPAP, systemic and pulmonary vascular resistance as well as arterial oxygenation remained constant as compared to baseline measurements in healthy piglets during the first experimental step: The wash-in of desflurane up to 1MAC did not affect general hemodynamics, respiratory mechanics, ventilation or global gas exchange.

**Parameter**	**Baseline**	**After DES wash-in**	**After DES wash-out**
MV (l/min)	5.33±0.25	5.5±0.4	5.5±0.2
etCO_2_ (mmHg)	43±1	45±4	45±4
*V*_T_ (ml)	260±8	277±20	273±11
PAW_peak_ (cmH_2_O)	18.9±0.1	18.3±0.2	18.7±0.1
PAW_mean_ (cmH_2_O)	9.5±0.1	9.4±0.1	9.6±0.1
*R*_tot_ (cmH_2_O/l/s)	6.6±0.2	5.9±0.3	5.7±0.4
paO_2_ (mmHg)	172±12	189±6	194±0
paCO_2_ (mmHg)	43±4	48±4	48±1
SaO_2_ (%)	100±0	99±1	99±1
pvO_2_ (mmHg)	43±1	41±1	43±4
pvCO_2_ (mmHg)	52±1	54±5	56±1
HR (1/s)	113±7	110±3	100±1
MAP (mmHg)	86±8	79±12	65±1
MPAP (mmHg)	21±1	21±2	20±1
CVP (mmHg)	6±1	6±1	7±1
CO (l/min)	4.3±0.2	3.9±0.2	3.6±0.2
PVR (dyn*s/cm^5^)	284±10	328±55	313±13
SVR (dyn*s/cm^5^)	1484±61	1497±308	1287±30

MV minute ventilation, *V*_T_ tidal volume; Paw airway pressure, *R*_tot_ total respiratory resistance, pa arterial partial pressure, SaO_2_ oxygen saturation in arterial blood, pv venous partial pressure, HR heart rate, MAP mean arterial pressure, MPAP mean pulmonary arterial pressure, CVP central venous pressure, CO cardiac output, PVR pulmonary vascular resistance, and SVR systemic vascular resistance.

**Table 2:** Cardiopulmonary variables prior to and after methacholine (MCh) administration by infusion (^#^*p*<0.05, in comparison with the healthy state, *n*=4). MCh given by infusion had significant effects on respiratory mechanics and gas exchange as well. Cardiac output was unaffected, and pvO_2_ did not change to any measurable degree. Peripheral vasodilation occurred as indicated by lower MAP and decreased SVR. There was a threefold increase of total respiratory resistance (*R*_tot_), and the airway pressure (PAW_peak_) were nearly doubled (*p*<0.05). In addition, MCh infusion caused a fall in the paO_2_/FIO_2_ ratio (*p*<0.05). The effects of MCh were not affected by desflurane uptake up to 1MAC during the second wash-in/wash-out period (^#^*p*<0.05 in comparison with healthy piglets at baseline).

**Parameter**	**During MCh infusion**	**After Des washin with MCh**	**After Des washout with MCh**
MV (l/min)	5.76±0.4	6.12±0.43	5.63±0.22
etCO_2_ (mmHg)	42±2	41±4	41±4
*V*_T_ (ml)	263±8	283±9	256±9
PAW_peak_ (cmH_2_O)	30±3.7^#^	27.7±1.4^#^	30.9±4.3^#^
PAW_mean_ (cmH_2_O)	12.0±0.9^#^	11.5±0.4^#^	11.9±0.9^#^
*R*_tot_ (cmH_2_O/l/s)	20.1±2.4^#^	16.8±0.9^#^	19.7±4.3^#^
paO_2_ (mmHg)	103±59^#^	95±39^#^	87±36^#^
paCO_2_ (mmHg)	48±2	50±5	49±4
SaO_2_ (%)	98±1.5	94±8 ^#^	91±7^#^
pvO_2_ (mmHg)	37±2 ^#^	35±3 ^#^	37±1 ^#^
pvCO_2_ (mmHg)	61±3^#^	61±6^#^	60±7^#^
HR (1/s)	103±17	105±26	115±18
MAP (mmHg)	61±7 ^#^	57±5 ^#^	67±6
MPAP (mmHg)	23±2	24±2	26±2 ^#^
CVP (mmHg)	7±1	10±2 ^#^	10±3 ^#^
CO (l/min)	3.6±0.5	3.9±1.2	4.2±0.5
PVR (dyn*s/cm^5^)	386±26 ^#^	312±71	333±89
SVR (dyn*s/cm^5^)	1197±160^#^	987±201^#^	1090±169^#^

MV minute ventilation, *V*_T_ tidal volume; Paw airway pressure, *R*_tot_ total respiratory resistance, pa arterial partial pressure, SaO_2_ oxygen saturation in arterial blood, pv venous partial pressure, HR heart rate, MAP mean arterial pressure, MPAP mean pulmonary arterial pressure, CVP central venous pressure, CO cardiac output, PVR pulmonary vascular resistance, and SVR systemic vascular resistance (Fig. 1).

**Table 3:** Median MIGET parameters and IQR prior to and after methacholine administration by infusion (*p<0.05, in comparison with the baseline healthy state). The quality of data was high as evidenced by low remaining sum of squares (RSS). MCh infusion and DES inhalation up to 1 MAC increased dead space and shunt but reduced perfusion to normal V̇A/Q̇.

**Variable**	**Healthy lung**	**MCh infusion**	**MCh infusion +DES**
**Alveolar ventilation**			
%V in low V̇_A_/Q̇	0 (0)	0 (0)	0 (0)
%V in normal V̇_A_/Q̇	57.3(16.1)	54.5(3.5)	44.4(23)
%V in high V̇_A_/Q̇	4.7 (7.2)	0 (0)	2.8 (8.4)
VD/VT (%)	38 (9)	45.4 (3.5)	52.9(14.7) *
mean of V_A_	1.5 (0.32)	1.03(0.02)	1.28(10.3)
**Pulmonary perfusion**			
Shunt (%)	1.6 (0.7)	4.8 (3.2)*	8.3 (3.2) *
%Q in low V̇_A_/Q̇	0 (0)	0 (0)	2.9 (8.8) *
%Q in normal V̇_A_/Q̇	97.7 (0.8)	95.3 (3.2)	84 (19.4) *
%Q in high V̇_A_/Q̇	0.2 (0.6)	0 (0)	3.8 (11.5) *
mean of Q	1.02(0.36)	0.79(0.05)	1.2 (0.74)
**Global parameters**			
log SDQ	0.5 (0.1)	0.5 (0.1)	0.9 (1.2)
log SDV	1 (0.5)	0.5 (0.1)	0.6 (0.3)
**Accuracy**			
RSS	2.7 (1)	1.3 (1)	3.3 (2.6)

A alveolar, V ventilation, Q perfusion, VD/VT dead space, and RSS remaining sum of squares.
